# Supplementary material for: Exploration of N6-Methyladenosine Profiles of mRNAs and the Function of METTL3 in Atherosclerosis
Source: Cells. 2022 Sep 24;11(19):2980. doi: 10.3390/cells11192980 (PMC9563305; doi:10.3390/cells11192980)
Supplement: Supplementary file 1 [file cells-11-02980-s001.zip › Supplementary Table S1.pdf]

Supplementary Table S1. The information from RNA-Seq and meRIP-Seq of ten hub genes identified by CytoHubba.

Gene	CytoHubba	RNA-Seq			MeRIP-Seq			
	MCC	Regulation	Fold change	*P* value	Regulation	Peak length	Fold change	*P* value
IL6	1.60E+07	down	-2.01	3.29E-02	/	/	/	/
IL1β	1.60E+07	down	-2.36	1.50E-04	down	69	2.40	2.81E-05
MMP3	1.58E+07	up	2.40	1.50E-04	up	101	2.50	1.74E-05
MMP1	1.53E+07	up	2.65	5.00E-05	up	185	2.26	3.70E-08
					down	403	2.88	3.93E-06
IGFBP3	1.52E+07	down	-19.38	1.00E-04	down	302	4.21	1.27E-03
					down	572	4.18	2.11E-10
LCN2	1.40E+07	down	-6.37	5.00E-05	down	137	4.40	4.13E-09
COL1A1	1.38E+07	down	-13.09	5.00E-05	down	348	2.11	2.39E-03
					down	219	4.68	4.39E-09
GDF15	1.37E+07	down	-3.17	5.00E-05	down	745	4.13	3.02E-10
					down	65	2.57	4.90E-05
TGFβI	1.28E+07	down	-2.49	3.00E-04	/	/	/	/
					down	1030	5.76	2.72E-09
					down	132	3.88	1.61E-08
					down	377	4.22	1.12E-10
SMAD3	1.06E+07	down	-2.73	5.00E-05	down	399	4.67	1.63E-10
					down	323	3.65	9.82E-09
					down	379	6.42	4.11E-11
					down	219	5.79	1.59E-11
