# Supplementary figures and images for: Exploration of N6-Methyladenosine Profiles of mRNAs and the Function of METTL3 in Atherosclerosis
Source: Cells. 2022 Sep 24;11(19):2980. doi: 10.3390/cells11192980 (PMC9563305; doi:10.3390/cells11192980)

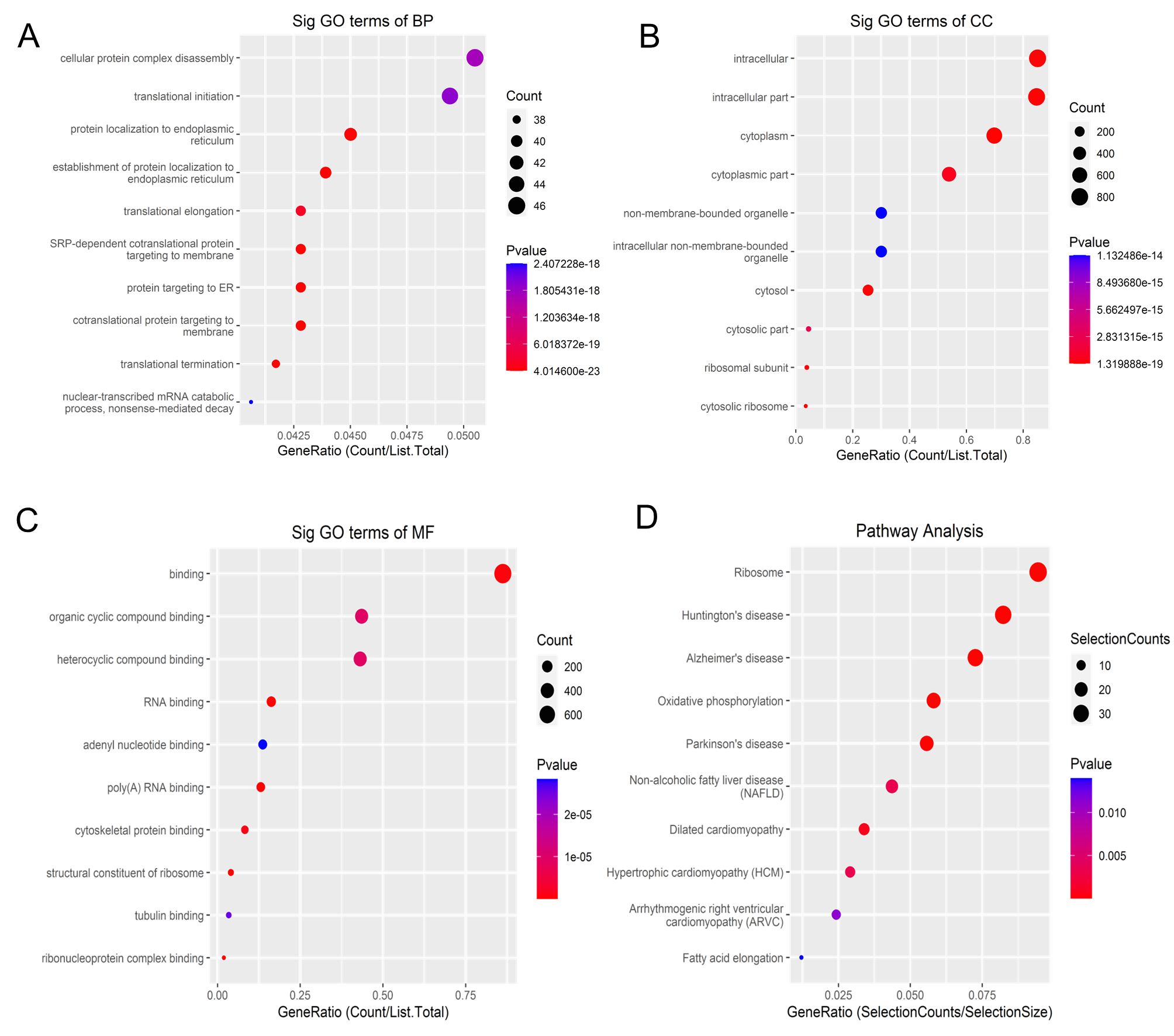

Supplement: Supplementary file 1 [file cells-11-02980-s001.zip › Supplementary Figure S1.tif]
